# Overview of Microvascular Angina Pectoris and Discussion of Traditional Chinese Medicine Intervention

**DOI:** 10.1155/2022/1497722

**Published:** 2022-01-05

**Authors:** Linghua Yu, Xiaoyan Lu, Chenxi Xu, Tong Li, Yanling Wang, Anxiang Liu, Yubi Wang, Li Chen, Huangyu Xu

**Affiliations:** ^1^Beijing University of Traditional Chinese Medicine, Beijing, China; ^2^China-Japan Friendship Hospital, Beijing, China

## Abstract

Previous research and treatment of coronary heart disease mostly focused on the large epicardial vessels, with limited research on the small endocardial coronary arteries or arterioles that could not be detected by coronary angiography, especially microvascular angina caused by microvascular stenosis or microcirculation dysfunction. Conventional Western medicine therapies have no specific efficacy, but traditional Chinese medicine has significant advantages in this regard. In particular, traditional Chinese medicine of supplementing Qi and activating blood circulation protects the vascular endothelium, relaxes coronary microvessels, reduces myocardial no-reflow after ischemia-reperfusion, increases myocardial hypoxia tolerance, constrains the aggregation of platelet, and increases the rate of blood flow. Moreover, these treatments can significantly improve patients' symptoms through multitarget comprehensive intervention. Here, we analyzed the pathogenesis of microvascular angina pectoris, the treatment status of modern medicine, and the research on the multitarget intervention of traditional Chinese medicine to provide new research ideas for correctly identifying the role of coronary microcirculation in coronary artery disease to solve clinical problems and prevent cardiovascular events.

## 1. Introduction

Previous studies [1] on coronary heart disease have mostly focused on large epicardial vessels, while studies on small endocardial coronary arteries are limited. Coronary angiography can only show vessels with a diameter of more than 500 *μ*m, usually epicardial vessels, while small vessels cannot be detected by coronary angiography. Therefore, interventional therapy can only solve the stenosis of large vessels and not the stenosis and dysfunction of small arterioles and microarteries. Furthermore, arterioles, microarteries, and venules together constitute the microcirculation of the coronary artery, which is the primary resistance vessel bed and myocardial metabolism site of the coronary artery and the main (95%) contributor to coronary resistance [2]. Therefore, in addition to the study of epicardial coronary artery disease, it is also important to pay close attention to the changes in coronary microvascular structure and function under pathological conditions. However, clinical research on coronary microvascular is limited and outdated. Further strengthening the research on coronary microvascular and microcirculation will improve the understanding of coronary heart disease and the formulation of prevention and treatment strategies of cardiovascular diseases and reduce the occurrence of cardiovascular events [3].

## 2. Research Overview of Microvascular Angina Pectoris

### 2.1. Coronary Microvascular Abnormalities

Even if there is no obvious stenosis of large blood vessels in clinical, an increase in microcirculation resistance can also lead to insufficient myocardial perfusion and ischemic heart disease events. A clinical study on the evaluation of symptoms of myocardial ischemia in the United States [3] demonstrated that as many as 50% of women and 17% of men with chest pain as the main complaint were confirmed to have normal coronary artery or only mild stenosis. In 1973, this group of symptoms with normal coronary angiography, positive results of electrogram and/or exercise load test, and typical exertional angina pectoris were defined as cardiac syndrome X [4] Recently, many studies [5] have shown that this syndrome is caused by myocardial ischemia due to a reduction in the reserve capacity of arterial dilation or coronary artery contraction. Therefore, it is considered that the lesion is located in the anterior arteriole, which cannot be detected by current coronary angiography technology and is termed microvascular angina (MVA). This concept was first proposed by Cannon and other experts [6, 7] in 1988 and may be related to microvascular abnormalities of the coronary artery.

The anterior arteriole is the main functional site for regulating myocardial blood perfusion. When the regulatory mechanism of anterior arterioles is unbalanced due to local nerve and body fluid factors or abnormal contraction of blood vessels distributed in sheets, the release of adenosine from distal local myocardial tissue will increase due to ischemia, which acts on afferent nerves and causes chest pain. Simultaneously, the arterioles regulated by metabolism expand and the intraluminal pressure decreases, resulting in further narrowing of anterior arterioles and arteriolar branches. The continuous increase in adenosine concentration in myocardial tissue leads to chest pain, which cannot be relieved [8]. Patients [9] with extensive stenosis or obstruction of the anterior arterioles will have decreased coronary flow reserve and corresponding changes of myocardial ischemia upon clinical examination. In contrast, patients [9] who have small-scale stenosis or obstruction of anterior arterioles, and no chest pain symptoms, often have no positive findings on various examinations. This explains why the symptoms of chest pain in patients with microvascular angina pectoris are serious, while the manifestations of myocardial ischemia and the left ventricular functional impairment are mild. Clinically, although some patients with acute myocardial infarction recanalize the coronary artery at the infarct site through revascularization treatment, the symptoms cannot be fundamentally improved because there is no reflow phenomenon in the microcirculation of the coronary artery at the infarct site [10, 11]. Research [12] has shown that 82.6% of patients have abnormal myocardial perfusion even if the thrombolysis in myocardial infarction (TIMI) classification of the patient is grade 3 after PCI. Even with the best drug treatment, nearly 50% of patients still have angina pectoris. Although some patients with myocardial infarction have coronary artery occlusion, the myocardial microcirculation in the corresponding area can be fully perfused through collateral circulation, so that symptoms in patients with myocardial infarction (MI) can be alleviated and the quality of life can be improved [13]. Therefore, microvascular reperfusion and the improvement of myocardial microcirculation are prerequisites to ensure myocardial survival after myocardial infarction.

### 2.2. Coronary Microcirculation Dysfunction

Coronary microcirculatory dysfunction (MCD) is currently considered [14] to be important in the pathogenesis of MVA and an independent predictor of adverse cardiovascular events. Beltrame et al. [15] stated that MCD is a disorder of coronary blood flow response caused by abnormal coronary microvascular resistance, resulting in insufficient myocardial perfusion and ischemia, which cannot be explained by epicardial coronary artery disorder. The coronary circulation system is responsible for providing oxygen and nutrients to cardiomyocytes, which is the coordination system between the volume vessel (with a diameter of 500 *μ*m–5 mm epicardial coronary artery) and the resistance vessel (with a diameter of <500 *μ*m). These tiny coronary arteries, which cannot be directly observed by angiography, constitute coronary microcirculation; the aforementioned provides important vascular resistance under normal conditions and determines the blood flow, redox, inflammation, agglutination, and other reactions of the coronary artery through metabolic regulation to meet the oxygen supply to the myocardium and myocardial perfusion [14]. Because MCD is deemed the key pathogenesis of patients with symptoms and signs of myocardial ischemia without obstructive coronary artery disease, correctly identifying the role of coronary microcirculation in coronary artery disease can lead to more accurate diagnosis and appropriate corresponding treatment measures for patients with ischemic cardiomyopathy.

At present, the main methods to evaluate myocardial microcirculation [10, 16] include myocardial contrast echocardiography, positron emission tomography, magnetic resonance imaging, and ultra-high-speed spiral computed tomography (CT). Myocardial contrast echocardiography is primarily used to quantitatively analyze myocardial blood perfusion, judge myocardial survival, judge coronary collateral circulation, measure coronary blood flow reserve, and evaluate the efficacy of interventional therapy or coronary artery bypass grafting. Furthermore, positron emission tomography, magnetic resonance imaging, and ultra-high-speed spiral CT are used to evaluate coronary microcirculation and demonstrate the presence of endocardial myocardial hypoperfusion during an attack of microvascular angina pectoris.

Under physiological conditions, the coronary microcirculation forms a mutually restrictive balance regulation mechanism by myogenic, blood flow-dependent, and metabolic contraction and expansion of coronary arterioles to maintain normal blood perfusion. Coronary microcirculation is an important factor in the development of coronary heart disease. Smoking, the elderly, hyperlipidemia, diabetes, and mental stimulation can all affect the function of microcirculation in small coronary artery endothelial cells [17, 18].

## 3. Pathophysiological Mechanism of MVA Induced by MCD

### 3.1. Decreased Coronary Flow Reserve

Current studies [14] believe that angina pectoris in patients with MVA is related to myocardial ischemia, and the reduction in coronary flow reserve (CFR) caused by MCD may be the main cause of myocardial ischemia [19]. Microvascular diastolic and systolic dysfunction increases the resistance of the coronary artery and often shows a poor response to vasodilation stimulation and enhanced vasoconstriction response. This results in abnormal arteriolar blood flow reserve, abnormal myocardial oxygen uptake dynamics in local uncoordinated areas, and imbalance between local or whole oxygen supply and blood flow, ultimately leading to myocardial ischemia [20].

### 3.2. Microvascular Endothelial Cell Dysfunction

Coronary microvascular endothelial dysfunction (CED) is an important link in the occurrence and development of MVA. Normal coronary endothelial cells can not only act as a mechanical barrier but can also receive and transmit information. In addition, they also have endocrine and metabolic functions and control and release a series of vasoactive substances, such as nitric oxide, prostacyclin, and endothelin. Angiotensin II is used to maintain the steady state of vasomotor and contraction and achieve the balance of myocardial perfusion and oxygen supply [21]. Maintaining the normal function and structural integrity of the vascular endothelium is of great significance to the smoothness of the vascular wall and the patency of blood flow. Endothelial cell damage can be caused by, among others, smoking, dyslipidemia, hypertension, and diabetes. Rubinstein and others [22] stated that CED plays an important role in the pathophysiology of MVA and leads to an independent disadvantage in judging the prognosis. The conclusion is supported by the research of Halcox et al. [23] who considered that the response of coronary microvessels to acetylcholine is related to the survival rate of patients. From the perspective of obstructive and nonobstructive coronary artery diseases, CED is the basis of the incidence and progression of coronary heart disease. Patients with MVA have significant endothelial dysfunction, which has been unanimously confirmed [24] by many studies, mainly manifested in the reduction of nitric oxide synthesis and reserve capacity and the significantly increased endothelin level. This endothelial dysfunction is not only demonstrated by the detection of endothelial-related vasoactive factors in the peripheral venous blood and coronary venous blood but also in the detection of various endothelial-dependent vasomotor functions.

### 3.3. Microvascular Arteriosclerosis

Because the diameter of the small coronary artery exceeds the resolution of angiography, there are no effective research technical means to confirm whether microvascular lesions occur based on coronary arteriosclerosis or are caused by microvascular stenosis. Supporting evidence [25] related to atherosclerosis is as follows: first, most patients with microvascular angina pectoris are complicated with atherosclerosis of the large coronary artery, but without significant stenosis, and the probability of vascular intervention in this area in the future is very high; second, there are many risk factors of atherosclerosis. Simultaneously, actively controlling the risk factors of atherosclerosis can improve the symptoms of myocardial ischemia (e.g., chest tightness and chest pain).

### 3.4. Patients with Inflammatory Factors

Patients with microvascular angina pectoris often have increased levels of various inflammatory factors [26], especially in the active period of the disease. The inflammatory response plays an important role in the occurrence and development of coronary atherosclerosis [27]. Inflammatory factors promote vascular endothelial proliferation, intimal thickening, and vascular remodeling. Additionally, inflammatory factors are directly involved in the inflammatory response, damage to the vascular endothelium, reduced release of nitric oxide and prostacyclin from endothelial cells, and weakened activity of endothelial cells. Moreover, the activation of endothelial cells, macrophages, and polymorphonuclear leukocytes releases human endothelin (ET), an inflammatory factor, and immune-like activators, resulting in abnormal endothelial systolic and diastolic function [28]. High-sensitivity C-reactive protein is the most studied inflammatory marker of cardiovascular disease [29], and its level can rapidly increase to more than 1000-fold in the inflammatory response stage [30].

### 3.5. Neurological Disorders

The imbalance of cardiac autonomic nerve function changes coronary artery microcirculation, which is considered to be related to MCD [31]. In particular, in the resting state, the increased release of norepinephrine from cardiac nerve endings not only enhances the microcirculation vasoconstriction response but also increases the sensitivity of resistance arterioles to vasoconstrictor stimulation, resulting in the occurrence of microcirculation dysfunction [32].

Among the above mechanisms, CED is considered the primary pathogenesis of MVA caused by MCD. Endothelial cells usually regulate blood flow by releasing diastolic and systolic factors, which act on the coronary smooth muscle and change the lumen diameter and blood flow, resulting in chest pain symptoms and ST segment changes. Coronary endothelial cell disorder causes excessive secretion of systolic factors and insufficient secretion of diastolic factors [33], which leads to systemic inflammation, hypercholesterolemia, and diffuse atherosclerosis. The microvascular endothelium is damaged, and the contraction of small blood vessels on coronary branches causes the accumulation of local myocardial metabolite adenosine, which acts on the afferent nerve of the heart and causes pain. In recent years, many studies [34–36] have shown that the metabolic disorder of various substances in serum is closely related to the pathogenesis of the disease. For example, high levels of leukocytes and high-sensitivity C-reactive protein are significantly correlated with endothelial dysfunction, while high plasma homocysteine can cause endothelial dysfunction and microcirculatory ischemia.

## 4. Prevention and Treatment of MVA and Intervention Ideas of Traditional Chinese Medicine

### 4.1. Overview and Problems with MVA Prevention

MVA is a recent research hotspot, and its pathogenesis includes many of the aspects mentioned above. Therefore, drug intervention should target multiple pathways, including the microvascular endothelium, microcirculation, inflammatory factors, platelet function, and myocardial and vascular function and regulation. At present, Western medicine has no specific therapeutic drugs for MVA. The existing drugs for treating coronary heart disease have a single mechanism, which can only intervene during disease unilaterally and not be adjusted from the whole mechanism. For example, traditional nitrates [36, 37] can only dilate coronary vessels with a diameter greater than 200 *μ*m. The thinner the vessels, the smaller the dilation effect. Although calcium channel blockers [37, 38] can prevent abnormal contraction of microvessels, nearly one-third of patients still have chest pain symptoms. Statins [38–40] can stimulate and upregulate the activity of endothelial nitric oxide synthase (NOS) to protect the endothelium or reduce oxidative stress to increase the bioavailability of nitric oxide. Statins can also improve endothelial function by inhibiting the expression of endothelin-1 (ET-1). Studies [40, 41] have shown that rosuvastatin can quickly improve coronary microvascular function in patients with acute myocardial infarction before and after interventional therapy. Angiotensin-converting enzyme inhibitors (ACEIs) and angiotensin receptor blockers (ARBs) improve coronary resistance and endothelium-derived relaxation by increasing NO bioavailability [41, 42]. Nicorandil [42–44] can alleviate the symptoms of chest pain, normalize ST-T changes, and improve coronary blood flow in patients with MVA, although it has side effects including headache and oral ulcer. Tirofiban [44–46] can prevent the formation of microthrombosis caused by platelet aggregation and improve microvascular endothelial function and coronary microvascular blood flow in patients with MVA. Its application in patients without reflow after intervention for acute coronary syndrome has attracted widespread attention. However, it has been reported that severe thrombocytopenia can occur during an emergency interventional treatment of acute myocardial infarction combined with tirofiban [46, 47].

### 4.2. TCM Intervention Ideas of MVA

MCD is an important pathogenesis of MVA and an independent predictor of adverse cardiovascular events. The dysfunction of coronary microvascular endothelial cells is considered [48] to be the primary pathogenesis of MVA caused by MCD. Therefore, grasping the importance of MCD for the occurrence, development, curative effect, and prognosis of coronary heart disease will be of great significance in the clinical treatment of MVA. Western medicine currently lacks specific drugs to treat this disease. Additionally, the level, link, and route of Western medicine as well as the drug resistance and toxic and side effects caused by the high selectivity of drugs are other difficulties that remain to be solved.

Traditional Chinese medicine focuses on syndrome differentiation and treatment under the guidance of the overall concept. It aims to regulate the balance of Yin and Yang and to prevent diseases. For example, for MVA, traditional Chinese medicine can prevent cardiovascular events by multitarget comprehensive interventions, such as protecting the vascular endothelium, alleviating vasospasm, and improving microcirculation, with both anti-inflammatory and antiplatelet effects. Compared to Western medicine, the targeted effects of traditional Chinese medicine monomer or compound on diseases are relatively mild; this is reflected in the “microeffect,” which reduces the possibility of toxic side effects and drug residues while pursuing a long-term curative effect. Recently, traditional Chinese medicine has made remarkable achievements in the prevention and treatment of microvascular angina pectoris (Table 1) and has great development potential. Numerous pharmacological studies [49, 51] have shown that the effective active components of traditional Chinese medicine can protect vascular endothelial cells. For example, baicalin and berberine extracts of heat-clearing drugs play anti-inflammatory and protective roles in the vascular endothelium by regulating the specific expression of endothelial autophagy. Moreover, Qi-tonifying drug [50] extracts, such as ginsenoside, *Astragalus* flavone, *Astragalus* saponin, and *Astragalus* polysaccharide, and the extracts of blood-activating drugs [50], such as ligustrazine and tanshinone, and other Chinese medicine monomers [51], such as resveratrol, *Uncaria* total alkaloids, and curcumin, have protective effects on vascular endothelial cells.

The components of traditional Chinese medicine are complex, with various pharmacological activities and different combinations. Therefore, its treatment mechanism can be reflected in many aspects, which are embodied in “multiple causes.” Recently, many studies have shown that traditional Chinese medicine decoctions, Chinese patent medicines, and traditional Chinese medicine injections commonly used in clinics have beneficial effects in the prevention and treatment of MVA. For example, clinical observation results show that Baoyuan decoction [52], compound Wenban decoction [54], Huoxue Tongmai Yixin decoction [55], Huoxue Zhuyu decoction [56], and Xuefu Zhuyu decoction [58] can reduce serum TNF in patients with coronary slow flow microvascular angina pectoris-*α* and IL-6 to increase the level of serum NO and improve the vasomotor function of vascular endothelial cells. Moreover, Danshen decoction [53], Ginseng Siwu decoction [57], and Zhishi Xiebai Guizhi decoction [59] can significantly reduce the frequency of angina pectoris, improve clinical symptoms, and improve the quality of life of patients, while Yixintongbi decoction [60] can significantly increase the CFR value and has a significant curative effect in improving TCM syndromes.

Chinese patent medicine has the advantages of a definite curative effect, less toxic side effects, and convenient use for treating microvascular angina pectoris. Clinical data show that Tongmai Yangxin pill [68], Yixinshu capsule [69], puerarin injection [78], and Yuxintong capsule [76] can upregulate the active decoction of vasoactive drugs to maintain the diastolic function of coronary microvessels, effectively reduce vascular resistance, improve intravascular skin function, and reduce myocardial no reflow after vascular reperfusion. *Salvia miltiorrhiza* polyphenolate [70], compound *Salvia miltiorrhiza* dropping pills [71–73], Danhong injection [79, 80], compound *Salvia miltiorrhiza* dropping pills [71–73], and Shenhong Huazhuo Tongluo granule [77] prevent platelet aggregation, prolong thrombosis, and reduce blood viscosity; this can quickly alleviate the slow flow condition of the coronary artery, increase blood flow velocity, improve myocardial reperfusion, reduce adverse cardiac events, and improve prognosis. Moreover, Qishen Yiqi dropping pill [61–63], Shexiang Baoxin pill [64, 65], Tongxinluo capsule [66], Shenqi capsule [74], and Shexiang Tongxin dropping pill [75] can inhibit cardiomyocyte apoptosis, slow the process of myocardial fibrosis, promote angiogenesis, and improve cardiac microvascular function while reducing myocardial ischemia-reperfusion injury.

## 5. Conclusion

In conclusion, traditional Chinese medicine can restore the balance of the body through “multicause and microeffect,” integration and coordination, and comprehensive intervention to restore the balance of the body, representing significant advantages in the treatment of diseases and providing new ideas for the prevention and treatment of coronary heart disease. Additionally, by summarizing the relevant literature, we found some problems in the current research. Firstly, there are few clinical studies of traditional Chinese medicine in this area, particularly clinical studies with high-level evidence-based medicine. Most clinical studies have problems such as small sample size, short research cycle, insufficient attention to the prognosis of the disease, and limited follow-up cycle. Secondly, the current clinical diagnostic technology of microvascular angina pectoris mostly depends on the evaluation of microvascular function. Most of these tests are radioactive, invasive, and expensive. The clinical use rate is low, resulting in an insufficient diagnosis of patients with MVA and inconsistent diagnostic standards of research objects. Thirdly, many experimental drugs are self-made prescriptions, with diverse treatment methods, but there is a lack of unified consensus, and the efficacy evaluation standards of each family are not unified, which is not conducive to their widespread clinical promotion. We should comply with the development tendency of integrated traditional Chinese and Western medicine and pay close attention to the top-level design of clinical research while continuously improving clinical diagnostic technology.

Future research should consider large sample survey and syndrome differentiation as the premise and conduct a large sample, multicenter, and randomized controlled trials by combining prescriptions and syndromes and selecting a compound of traditional Chinese medicine as the research goal so as to better serve the advantages of “microeffect and multicause” and comprehensive intervention of traditional Chinese medicine. Additionally, paying attention to the follow-up of adverse drug reactions and outcome events and standardizing the clinical treatment methods and efficacy evaluation standards of traditional Chinese medicine will provide new research ideas to correctly identify the role of coronary microcirculation in coronary artery disease, thereby solving clinical problems and preventing and treating cardiovascular events.

## Figures and Tables

**Table 1:** Research on the prevention and treatment of microvascular angina pectoris by traditional Chinese medicine.

Category	Content
Single drug extract	Baicalin [49], ginsenoside [50], *Astragalus* flavone [50], *Astragalus* saponin [50], *Astragalus* polysaccharide [50]. ligustrazine [50], tanshinone [50], resveratrol [51], *Uncaria* total alkaloids [51], curcumin [51], and berberine [51]
Traditional Chinese medicine decoction	Baoyuan decoction [52], Danshen decoction [53], compound wendan decoction [54], Huoxue tongmai yixin decoction [55], Huoxue zhuyu decoction [56], Ginseng Siwu decoction [57], Xuefu zhuyu decoction [58], Zhishi xiebai guizhi decoction [59], and Yixin tongbi decoction [60]
Chinese patent medicine	Qishen Yiqi dropping pill [61–63], Shexiang baoxin pill [64, 65], Tongxinluo capsule [66, 67], Tongmai yangxin pill [68], Yixinshu capsule [69], Salvia miltiorrhiza polyphenolate injection [70], Compound salvia miltiorrhiza dropping pill [71–73], Shenqi capsule [74], Shexiang tongxin dropping pill [75], Yuxintong capsule [76], Shenhong huazhuo tongluo granule [77], Puerarin injection [78], and Danhong injection [79, 80]
